# The Effect of Hot-Melt Extrusion of Mulberry Leaf on the Number of Active Compounds and Antioxidant Activity

**DOI:** 10.3390/plants11223019

**Published:** 2022-11-09

**Authors:** Hyun-Bok Kim, Suji Ryu, Jong-Suep Baek

**Affiliations:** 1 National Institute of Agricultural Sciences, RDA, Wanju 55365, Korea; 2 Department of Bio-Health Convergence, Kangwon National University, Chuncheon 24341, Korea; 3 Department of Herbal Medicine Resource, Kangwon National University, Samcheok 25949, Korea; 4 BeNatureBioLab, Cuncheon 24206, Korea

**Keywords:** mulberry leaf, hot-melt extrusion (hme), water solubility, rutin, isoquercetin, antioxidant activity

## Abstract

The aim of this study is to compare the functions of the physiologically active compounds of three types of mulberry leaf by cultivar, and to confirm the changes using hot-melt extrusion (HME−ML). The active components of mulberry leaf were analyzed using the HPLC system, and total phenolic content (TPC), total flavonoid content (TFC), and antioxidant activity were measured. Among the three varieties, the highest contents of rutin and isoquercetin were detected in Cheongil, of TPC in Cheongol, and of TFC in Cheongil. It was confirmed that this bio-accessibility was increased in HME−ML compared with the control. The DPPH radical scavenging activity of Cheongol showed greater antioxidant properties, and HME showed improvement in the antioxidant properties of all mulberry leaves. These results suggest that the application of HME technology can improve the biological activities of mulberry leaf.

## 1. Introduction

Mulberry leaf (*Morus alba* L.) has been used in traditional medicine and is an edible plant used to grow silkworms [1]. It is currently used to make diet foods or to develop products, such as fortified beverages, yogurts, and teas [2]. Mulberry leaf is used in traditional Chinese medicine to treat diabetes and hyperlipidemia and contains secondary metabolites, such as flavonoids, organic acids, alkaloids (1-deoxynojirimycin, fagomine), and polyphenols [3,4,5]. Mulberry leaf includes the effects of lowering blood sugar, antioxidant, anti-inflammatory, high-density lipoprotein–cholesterol increase, and low-density lipoprotein–cholesterol reduction [6,7,8]. A previous study found that the functional components of mulberry leaves differ between varieties [9]. Therefore, it is important to select suitable mulberry leaves through activity studies according to variety.

The active components of the mulberry leaf include rutin, isoquercetin, and quercetin [4,10]. Isoquercetin and rutin are flavonoids, which are a family of polyphenolic compounds. The content difference between total phenol content (TPC) and total flavonoid content (TFC) may be caused by growth environment, contamination, and pathogens [11,12]. The water solubility of rutin and isoquercetin was 0.125 g/L and 0.095 g/L. Although many biological activities have been revealed due to poor water solubility, they have the disadvantage of showing low bioavailability and absorption when applied to food development [13,14].

Hot-melt extrusion (HME) has proven to be a successful technique in drug delivery systems (DDS) and several applications through properties such as the improved solubility of poorly soluble compounds, targeting, drug delivery, and the preparation of nanoparticles [15]. HME is widely used in the food industry and pharmaceuticals. HME converts a mixture into having certain properties, such as uniform shape and density, by forcing the mixture through a die [16]. The principle of HME is to induce melting and further solubilization and fusion by applying heat and friction (interparticle friction, sample and wall friction, sample, and screw friction) to the mixture [17]. The advantage of HME is that it is an eco-friendly technology that does not use organic solvents, improves the dispersal of the drug and the solubility of the poorly soluble drugs, and increases bioavailability. However, the disadvantage of HME is that it requires high temperature and must be stable under thermal decomposition [18]. Charunuch et al. compared antioxidant activity and TPC according to the various conditions of HME by mixing mulberry leaf and instant cereal beverage powder. As the content of mulberry leaf in the mixture increased, antioxidant activity and TPC increased [19].

The HME increases solubility by producing a stable amorphous solid dispersion and increased energy form through a combination of processing and excipients [20]. Whey protein isolate (WPI) is receiving attention as a food biopolymer in the food industry, and heat treatment above 60 degrees causes the denaturation of WPI, exposing many hydrophobic functional groups on the surface of protein particles [21]. Among natural emulsifiers, lecithin is an amphiphilic surfactant that can bind proteins through hydrophobic interactions [22]. Lecithin, used as a surfactant, improves the solubility of active ingredients by improving solubility, and is also used as a plasticizer for the matrix [23]. During the HME process, the addition of plasticizer could decrease processing temperature and achieve higher dispersion [24,25]. Citric acid was used as a pH adjuster to control drug release in hydroxypropyl methylcellulose (HPMC) matrix tablets [26] and was used as a solid-state plasticizer when preparing a solid dispersion through HME [27,28,29]. Ascorbyl palmitate, an amphiphilic synthetic derivative of ascorbic acid, is used as a natural antioxidant in the food industry [30,31] and can be used as an emulsifier [32]. In HME studies with Moringa oleifera Lam, ascorbyl palmitate was used as a plasticizer [33]. Vitamin C is widely used in food as a naturally occurring antioxidant and has been used as a plasticizer for biodegradable polymers [34]. Ascorbyl palmitate, Vitamin E, and WPI were mixed and coated to protect peanuts from lipid oxidation [35].

The purpose of the study was to improve the active compound water solubility of three mulberry leaf varieties through the HME. The mulberry leaf and HME-mulberry leaf (HME−ML) were compared through high-performance liquid chromatography (HPLC), antioxidant, and TPC and TFC analysis.

## 2. Materials and Methods

### 2.1. Materials

The materials used in this study are three types of mulberry leaf grown in Korea, namely the Cheongol, Iksu, and Cheongil varieties. To avoid any effect from pedoclimatic factors, the three varieties were grown and collected at the same place, the National Institute of Agricultural Sciences, (Wanju, Korea) in early May. Acetic acid was purchased from Duchefa (Haarlem, The Netherlands). Acetonitrile (ACN) was purchased from Fisher (A9984, Waltham, MA, USA). Folin–Ciocalteu’s phenol reagent (F9252), gallic acid (G7384), and quercetin (Q4951) were purchased from Sigma–Aldrich (St. Louis, MO, USA). Sodium carbonate was purchased from Daejung (7541-3300, Siheung, Korea). Potassium acetate was purchased from TCI (P2786, Tokyo, Japan). Aluminum chloride hexahydrate, 2,2-diphenyl-1-picrylhydrazyl (DPPH) (044150), and L-ascorbic acid (011188) were purchased from Alfa Aesar (Ward Hill, MA, USA).

### 2.2. Extraction Method

The weights of the mulberry leaf and HME−ML were all calibrated to 1 g (100%) and weighed. Deionized water (D.W.; 50 mL) was used as an extraction solvent. An ultrasonic cleaner (UCP-20, JeioTech Co., Ltd., Daejeon, Korea) was used for ultrasonic extraction (40 °C, 30 min). The extract was centrifuged at 3000 rpm, and the supernatant was filtered by Whatman filter paper No. 6 (Cytiva, Marlborough, MA, USA) and recovered by rotary pressure concentration (Eyela Co., Ltd., Tokyo, Japan).

### 2.3. Preparation of HME-ML

After mixing the mulberry leaf and each additive, the HME process (STS-25HS tween screw extruder, Hankook E.M. Ltd., Pyoung-Taek, Korea) was performed. As additives, whey protein isolate, soy lecithin, vitamin C, vitamin E 50%, citric acid, and ascorbyl palmitate were used, after mixing in predetermined ratios (Table 1). The ratio of additives in HME processing was carried out under the same conditions to compare the activity according to the varieties of mulberry leaves. The processing temperature was fixed at 100 °C. The conditions of HME process were 40~50 bar of pressure and 50 rpm of screw speed. The HME-ML was used for subsequent experiments after drying.

### 2.4. HPLC Analysis

The mulberry leaf was extracted with distilled water to prepare 1 mg/mL and analyzed. The calibration curve was prepared with rutin and isoquercetin (Sigma–Aldrich Co., St. Louis, MO, USA). The instrument used for HPLC analysis was a Simadzu LC-20AT HPLC system (Tokyo, Japan). Samples were filtered with a syringe filter (0.45 µm) before analysis. Water containing 0.5% acetic acid and acetonitrile (ACN) was used as a solvent. It proceeded according to the analysis conditions (Table 2).

### 2.5. Total Phenolic Content (TPC)

TPC was tested using the Folin–Denis method [36]. The concentration of the sample was 1 mg/mL, and in 20 µL of the sample, 100 µL of Folin–Ciocalteu’s phenol reagent and 80 µL of 7.5% sodium carbonate were added. Absorbance was measured at 760 nm using a microplate reader (Epoch, Agilent Technologies Inc., Santa Clara, CA, USA) at room temperature (RT) for 45 min in the dark. The standard curve was prepared at 20, 40, 60, 80, and 100 µg/mL using gallic acid, respectively, and a calibration curve was then prepared.

### 2.6. Total Flavonoid Content (TFC)

The TFC was tested using the Dowd method [37]. The concentration of the sample was 1 mg/mL, and after adding 60 µL of 95% ethanol, 4 µL of 10% aluminum chloride hexahydrate, 4 µL of 1 M potassium acetate, and 112 µL of D.W. to 20 µL of the sample, it was reacted at RT for 40 min. Absorbance was measured at 415 nm using a microplate reader. The standard curve was prepared at 20, 40, 60, 80, and 100 µg/mL using quercetin, respectively, and a calibration curve was then prepared.

### 2.7. Antioxidant Activity

Antioxidant activity was tested using 2,2-diphenyl-1-picrylhydrazyl (DPPH) [38]. Samples were prepared at 0, 1000, 2000, 3000, 4000, and 5000 µg/mL, and then 150 µL of 0.4 mM DPPH solution that was prepared in methanol was added to 5 µL of each sample, followed by reaction in the dark at RT for 30 min, and the absorbance was measured at 517 nm. L-ascorbic acid was used as a control, and a comparative experiment was performed after preparation at 0, 20, 40, 60, 80, and 100 µg/mL. DPPH radical scavenging activity was measured by IC_50_ (µg/mL), which is a concentration that reduces DPPH radical by 50%. The inhibition rate of the mulberry leaf was calculated using the following equation:

$$\mathrm{Inhibition}\;\left(\%\right)=\left(\mathrm{blank}\;\mathrm{absorption}-\frac{\mathrm{extract}\;\mathrm{sample}\;\mathrm{absorption}}{\mathrm{blank}\;\mathrm{absorption}}\right)\times 100$$

### 2.8. Statistical Processing

The results of repeated HPLC, TPC, TFC, and DPPH radical scavenging activity experiments using SAS 9.4 (SAS Institute Inc., Cary, NC, USA) were expressed as the mean ± standard deviation. Significant differences between samples were tested for significance at the Duncan’s Multiple Range Test (DMRT) 5% level (*p* < 0.05).

## 3. Results and Discussion

### 3.1. Analysis of Isoquercetin and Rutin Contents of Mulberry Leaf and HME-ML

**Figure 1:**
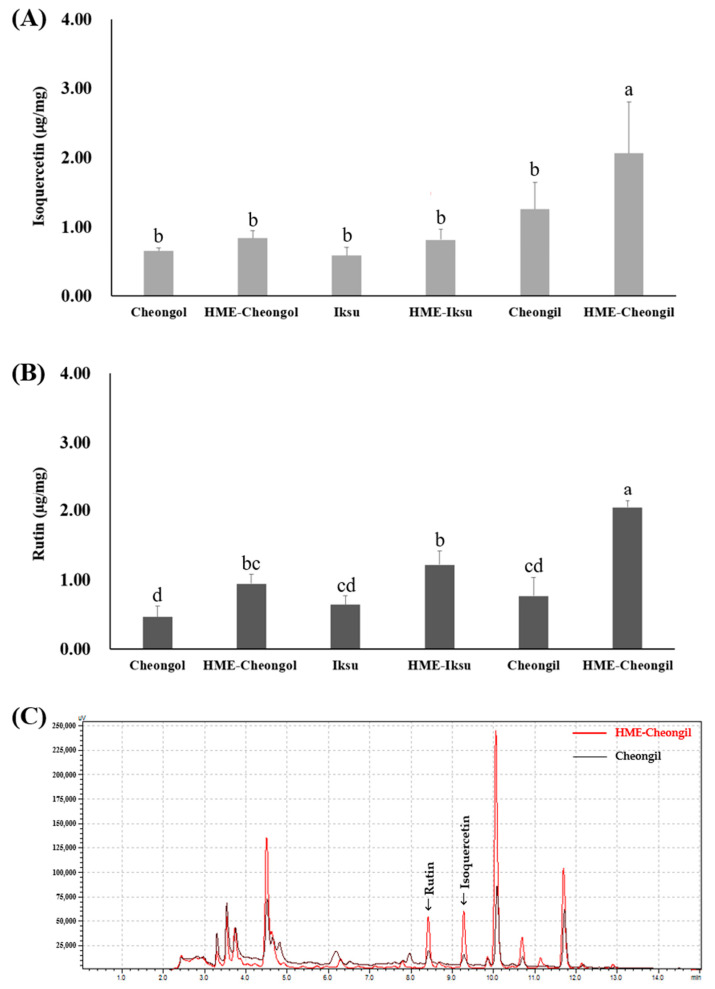
(**A**) Isoquercetin and (**B**) rutin contents of mulberry leaf and HME-ML extract; and (**C**) HPLC chromatogram of rutin and isoquercetin in Cheongil and HME-Cheongil extract. Data are expressed as mean ± standard deviation (*n* = 3). Means with different letters vary significantly with Duncan’s Multiple Range Test (DMRT) at 5% level (*p* < 0.05) within samples.

The isoquercetin and rutin content of mulberry leaf and HME−ML before and after HME showed different results in Figure 1. Cheongil had the highest isoquercetin content, and it was confirmed that there was a difference of about two times compared to other varieties. After HME processing, isoquercetin content was increased in all varieties, and HME−Cheongil showed the highest content in the HME formulation. The rutin content was the highest in Cheongil, and after HME processing, the rutin content was increased in all varieties, and HME−Cheongil showed the highest content in the HME formulation.

The rutin content of mulberry leaf extracted using methanol was reported to be the highest in Cheongol and the lowest in Choengil [39]. Rutin and isoquercetin are generally poorly soluble in water [14,40], and HME increases the solubility of liposoluble components [41,42,43]. Park et al. confirmed the increased rutin content through HME processing of buckwheat flour, and the rutin content increased as the mixed yeast content increased [44]. When lecithin and vitamin E were added in the HME processing of buckwheat and potato, the content of rutin was increased. In addition to rutin, the increase in phenol and flavonoid contents may be due to the encapsulation of active components through lecithin liposome formation [45]. The results show that through the HME process, the solubility of liposoluble components, rutin, and isoquercetin increased in water, resulting in an enhanced amount of hydrophobic active compounds.

### 3.2. Total Phenolic Content

The TPC results are presented using gallic acid as a standard. Among the three mulberry leaf varieties, Cheongol had the highest TPC content. In all mulberry leaf varieties, TPC was higher after HME, and HME−Cheongol showed the highest TPC content of 31.14 ± 4.63 mg/g (Table 3). High temperature and shear forces applied to phenolic compounds can form amorphous structures and lead to increased solubility through the destruction of cellular components [46]. An increase in TPC by HME was also reported in another study [45]. It was confirmed that for all mulberry leaf varieties, TPC was higher after extrusion than before extrusion. This suggests that when HME is applied to mulberry leaf, TPC is increased. This result could be explained as the increased water solubility of rutin and isoquercetin correlating with the total amount of phenolic content.

### 3.3. Total Flavonoid Content

The TFC results are presented using quercetin as a standard. Among the three mulberry leaf varieties, Cheongil had the highest TFC content, and in HME−ML, HME−Iksu showed the highest TFC content of 22.12 ± 3.9 mg/g (Table 3). It was confirmed that for all mulberry leaf varieties, TFC was higher after extrusion than before extrusion. This suggests that when HME is applied to mulberry leaf, TFC is increased. Wang et al. suggested that the total flavonoid solubility of Ginkgo biloba extract was increased through HME and could increase oral bioavailability [47].

### 3.4. Antioxidant Activity

The antioxidant activity of mulberry leaf extracts was compared by DPPH radical scavenging activity (Table 3). Among the three mulberry leaf varieties, Cheongol had the lowest IC_50_, and in HME−ML, HME−Cheongol showed the lowest IC_50_ of 4480.83 ± 35.63 µg/mL. HME−ML had a higher IC_50_ value than ascorbic acid, but all mulberry leaf varieties showed a lower IC_50_ value than non-HME−ML. The DPPH radical scavenging activity was increased after HME processing in all mulberry leaf varieties, and it was confirmed that the inhibition rate was increased in a concentration-dependent manner. This suggests that the application of HME to mulberry leaf increases antioxidant activity. Among the natural antioxidants, phenolic compounds, which are secondary metabolites produced by plants, are contained the most. The TFC and TPC in mulberry leaf have been found to have a significant effect on antioxidant activity [12]. *Phaseolus bulgaris* L. shows a difference in antioxidant activity depending on the cultivar, and the free radical scavenging activity was faster in the extrudate extract [48].

## 4. Conclusions

In this study, HME with proper additives enabled improved water solubility of rutin and isoquercetin. The isoquercetin content of the three kinds of the mulberry leaf was high in the order Cheongil > Cheongol > Iksu, and it was confirmed that the rutin content was high in the order Cheongil > Iksu > Cheongol. Through HME, it was confirmed that the content of isoquercetin and rutin in all mulberry leaves was increased, and through this, it was confirmed that the solubility of the active ingredient, which showed low water solubility, was improved. TPC was the highest in the order Cheongol > Iksu > Cheongil, and TFC was the highest in the order Cheongil > Cheongol > Iksu. In antioxidant activity using DPPH assay, Cheongol showed the lowest IC50 value in the order Cheongol < Iksu < Cheongil, and after HME processing, all mulberry leaves showed a decrease in IC50 value. HME-treated Cheongil was found to have the highest contents of rutin and isoquercetin. Moreover, it showed the highest antioxidant activity in the DPPH assay, suggesting that the correlation between the active compound and antioxidant activity contributed to the improvement of antioxidant capacity. The results provide information that different mulberry leaf varieties may be used for different purposes in the future. Furthermore, this suggests that HME can be used as a candidate to enhance the biological activities of the mulberry leaf.

## Figures and Tables

**Table 1:** The composition of HME−ML.

Chemical Formulation (%)	HME− Cheongol	HME− Iksu	HME− Cheongil
Mulberry leaf	79	79	79
WPI	10	10	10
Soy Lecithin	2.5	2.5	2.5
Vit C	2	2	2
Vit E 50	2	2	2
Citric acid	2	2	2
Ascorbyl palmitate	2.5	2.5	2.5
Total Ratio (%)	100	100	100

**Table 2:** The HPLC analysis conditions.

Column	YMC-ODS AM C18 (5 µm, 12 nm) 250 mm × 4.6 mm	
Detector	UV-VIS detector (356 nm)	
Solvent A	Water containing 0.5% acetic acid	
Solvent B	Acetonitrile (ACN)	
Flow rate	1 mL/min	
Oven	35 °C	
Injection volume	10 µL	
Time (min)	Gradient elution system	
	% A	% B
Initial	82	18
9	65	35
11	82	18
22	82	18

**Table 3:** Total phenolic content (TPC), total flavonoid content (TFC), and antioxidant activity by DPPH of mulberry leaf extract.

Sample	TPC	TFC	DPPH
	GAE·mg/g	QE·mg/g	IC_50_ (µg/mL)
Cheongol	14.48 ± 0.73 ^cd^	8.82 ± 0.35 ^b^	7880.56 ± 1084.82 ^b^
HME−Cheongol	31.14 ± 4.63 ^a^	19.68 ± 1.48 ^a^	4480.83 ± 35.63 ^d^
Iksu	11.96 ± 4.15 ^d^	8.77 ± 0.66 ^b^	8733.33 ± 196.392 ^a^
HME−Iksu	15.72 ± 0.58 ^cd^	22.12 ± 3.91 ^a^	4530.83 ± 51.30 ^d^
Cheongil	11.17 ± 4.31 ^d^	12.26 ± 1.12 ^b^	8963.33 ± 297.75 ^a^
HME−Cheongil	23.96 ± 5.78 ^ab^	22.02 ± 2.97 ^a^	5778.33 ± 235.39 ^c^
Ascorbic acid	-	-	167.49 ± 15.69 ^e^

Data are expressed as mean ± standard deviation (*n* = 3). Means with different letters vary significantly with DMRT at 5% level (*p* < 0.05) within samples.

## Data Availability

All data are available in the article.
